# The Heart–Brain Interplay in Multiple Sclerosis from Pathophysiology to Clinical Practice: A Narrative Review

**DOI:** 10.3390/jcdd10040153

**Published:** 2023-04-01

**Authors:** Matteo Zavarella, Andrea Villatore, Maria Assunta Rocca, Giovanni Peretto, Massimo Filippi

**Affiliations:** 1 Neuroimaging Research Unit, Division of Neuroscience, IRCCS San Raffaele Scientific Institute, 20132 Milan, Italy; 2 Faculty of Medicine and Surgery, Vita-Salute San Raffaele University, 20132 Milan, Italy; 3 Department of Cardiac Electrophysiology and Arrhythmology, IRCCS San Raffaele Scientific Institute, 20132 Milan, Italy; 4 Myocarditis Disease Unit, IRCCS San Raffaele Scientific Institute, 20019 Milan, Italy; 5 Neurology Unit, IRCCS San Raffaele Scientific Institute, 20132 Milan, Italy; 6 Neurorehabilitation Unit, IRCCS San Raffaele Scientific Institute, 20132 Milan, Italy; 7 Neurophysiology Service, IRCCS San Raffaele Scientific Institute, 20132 Milan, Italy

**Keywords:** multiple sclerosis, autoimmunity, cardiovascular disease, autonomic dysfunction, myocarditis, drug toxicity

## Abstract

Multiple sclerosis (MS) is a chronic neurological disorder characterized by inflammation in the central nervous system (CNS) that leads to neurodegeneration. The clinical course is highly variable, but its prevalence is rising worldwide, partly thanks to novel disease-modifying therapies. Additionally, the lifespan of people with MS is increasing, and for this reason, it is fundamental to have a multidisciplinary approach to MS. MS may be associated with cardiovascular diseases (CVD), but there is scarce attention on this issue. In particular, CNS is essential in regulating the autonomic system and heart activity. Moreover, cardiovascular risk factors show a higher prevalence in MS patients. On the other hand, conditions like Takotsubo syndrome are rare complications of MS. The parallelism between MS and myocarditis is also interesting. Finally, cardiac toxicity represents a not infrequent adverse reaction to MS drugs. This narrative review aims to provide an overview of cardiovascular complications in MS and their management to prompt further clinical and pre-clinical research on this topic.

## 1. Introduction

**Figure 1:**
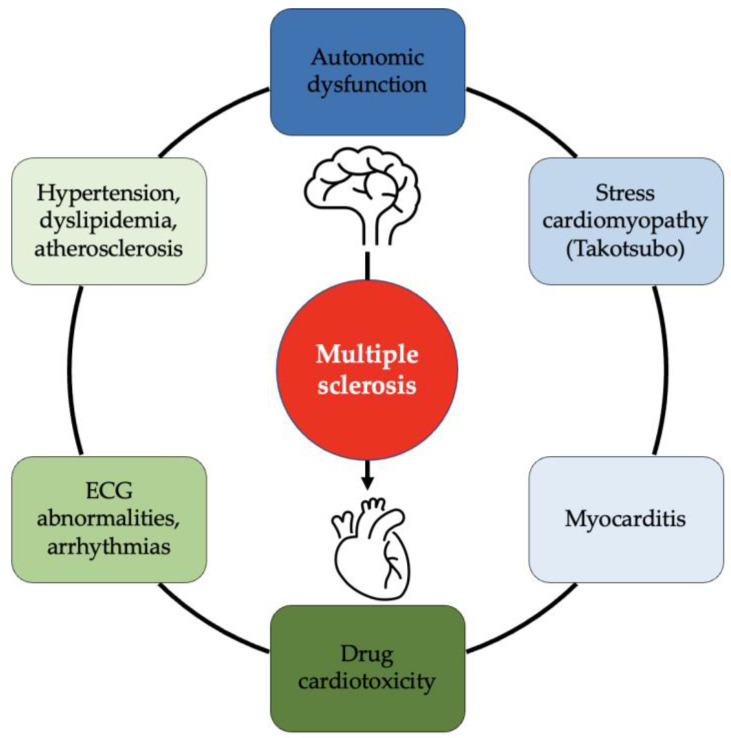
The heart–brain interplay in multiple sclerosis (MS). Cardiovascular manifestations may occur in MS patients. Autonomic dysfunction is an important cause of disability in MS patients. Cardiovascular risk factors, such as hypertension, dyslipidemia, and atherosclerosis, as well as arrhythmias, show high prevalence in MS patients. Cardiac toxicity represents a not infrequent adverse reaction of MS drugs. Finally, Takotsubo syndrome and myocarditis represent a rare complication of MS.

Multiple sclerosis (MS) is a chronic neurological disease characterized by inflammation and demyelination in the central nervous system (CNS), with these processes ultimately leading to neurodegeneration. The clinical course of MS is heterogeneous, but ineluctably patients accumulate a significant burden of clinical disability over time [1]. The current understanding of the pathophysiology is based on the participation of different immune cell types: T cells, B cells, but also CNS-resident microglia [2,3]. Consequently, MS pathophysiology involves inflammatory processes that develop both in the peripheral and the central compartment. Inflammation and pro-inflammatory cytokines are responsible for the damage of the CNS; they cause the dysregulation of the blood-brain barrier (BBB) and the trans-endothelial migration of activated leukocytes, leading to the loss of oligodendrocytes and to neuro-axonal degeneration mediated by activated immune cells. Macroscopically, the pathological process is observable as focal lesions, which occur throughout the CNS (brain, optic nerve, and spinal cord), involving both white and grey matter. In addition to plaques, normal-appearing tissues are extensively engaged in MS-related pathological processes [4]. Considering this pathological heterogeneity, the clinical manifestations of MS are highly variable and implicate abnormal functioning of both the autonomic and somatic nervous systems. Patients with MS experience impaired functional capacity and have a life expectancy of 5–7 years shorter than the general population [5]. Since the presence of comorbidity is associated with increased mortality in MS [6], identifying and treating adequately the comorbidities is fundamental to the optimal management and the improvement of the quality of life (QoL) of these patients [7]. The most common MS-unrelated causes of death in patients with MS are cancer, cardiovascular disease, and respiratory disease [8]. In this review, we will focus on heart diseases that can manifest in the course of MS. There is a main concern about the increased mortality due to cardiovascular disease in MS patients compared with the general population [9]. To better understand the cause of this phenomenon, we will discuss the current knowledge about three critical aspects that involve the interaction between the cardiovascular and nervous systems. First, the CNS has an important role in regulating the autonomic system; hence, altered autonomic functions observed in MS patients can directly affect heart activity. Heart alterations in this context may become clinically relevant or, more often, remain discrete, but they seem to correlate with inflammation and clinical progression [10]. Second, cardiovascular risk factors are surprisingly prevalent in MS. Compared with the general population, more physical limitations and a higher prevalence of hypertension, hyperlipidemia [11], adipose mass, and smoking exposure have been reported in MS [12]. All the conditions listed above are well-known risk factors for cardiovascular disease [13]. Moreover, MS determines a state of chronic inflammation, which is also an important cardiovascular risk factor [14]. Third, therapy for MS is constantly evolving, improving the survival of the patients. Innovative treatments can lead to great success in controlling MS activity, but the patients’ survival comes with new challenges when managing this chronic condition. As a matter of fact, cardiovascular comorbidities increase their incidence and prevalence along with the aging of the patients. Furthermore, the potential cardiotoxicity of certain chronic therapies should be carefully evaluated. The aim of this manuscript is to summarize the current knowledge (see Figure 1), discuss the open questions about MS and acquired heart disease, and give practical clinical advice. It is fundamental to increase awareness of the problem and stimulate research about this condition to understand and treat it better, thus ultimately leading to better care for MS patients.

### 1.1. Autonomous Nervous System

The simultaneous functioning of all the internal organs is critical for responding to environmental stimuli, maintaining homeostasis, and putting into action the complex reaction of human behavior. The goal of the autonomic nervous system (ANS) is to coordinate the internal organs in an involuntary way [15]. ANS is composed of cellular elements both inside and outside the CNS. ANS is able to receive information from the viscera, elaborate the information at the spinal cord and brainstem level, and send signals outside the CNS through effector fibers. The regulatory center of the ANS and the cortex can engage in a bidirectional relationship, especially in cases of response to external stimuli [16]. ANS also has a fundamental role in controlling heart activity, influencing cardiac muscle fiber contraction, and modifying cardiac output and blood pressure. The core of the central ANS is the central autonomic network (a connectome matrix between the insular cortex, amygdala, hypothalamus, periaqueductal grey matter, nucleus tractus solitarius, and ventrolateral medulla) [17], which modulates cardiac repolarization and heart rate, in addition to other autonomic functions [18]. ANS is functionally divided into two main branches, the sympathetic and the parasympathetic nervous system, that affect heart rate, contractility of heart fibers, and velocity of the electrical conduction heart system. Noteworthy, blood vessels are among the few structures that receive inputs just from the sympathetic nervous system. It is possible that the two branches of the ANS are not affected simultaneously in the same way by MS, but an earlier involvement of the sympathetic nervous system might occur [19]. In addition, the heart is provided with an intrinsic cardiac nervous system [20] that can transmit afferent signals to the brain [21]. Inputs deriving from the viscera gain significant importance in emotional regulation and perception of feelings. For this reason, an altered cardio-neural interplay should be investigated to look for fatigue, tiredness, and emotional imbalance experienced by patients with MS. Finally, the nervous system can indirectly influence cardiac functioning by regulating the level of circulating hormones, such as catecholamines and cortisol.

### 1.2. ANS in MS Patients

Dysfunction of the ANS represents an important cause of MS-related disability [22]. MS symptoms caused by ANS alterations are relevant for MS patients and involve several ANS-related systems. MS patients experience cardiovascular, urinary, sexual, intestinal, and sweating problems [23]. Cardiovascular autonomic dysfunction is reported in up to two-thirds of MS patients and can present with different severity [10]. It is currently accepted that cardiac autonomic dysfunction can result from demyelinating plaques in the brainstem [24,25]. For example, Saari and colleagues found that the volume of midbrain lesions and, to a lesser extent, the volume of hemispherical lesions were associated with cardiovascular dysfunction [26]. Autonomic dysfunction can also originate from secondary axonal loss in the spinal cord, as supported by a study that found a correlation between ANS dysfunction and spinal atrophy in MS patients [27]. Lesions located in critical areas for heart control constitute a risk factor for heart disease in MS patients and may cause the altered cardiac repolarization observed in this population [28,29]. The timing and the pathogenetic role of autonomic dysfunction in the progression of MS are not clearly understood. Assessing cardio-neural interaction through cardiac activity could be very informative for the prognosis and QoL of these patients. Outstandingly, the presence of postural orthostatic tachycardia syndrome (POTS) in patients with a clinically isolated syndrome (CIS) was a negative prognostic factor for evolution to definite MS [30]. The health of the cardio-neural system is easily monitored by several measures, such as heart rate responses to different maneuvers, heart rate variability recording, repolarization markers, myocardial contractility measure, and baroreceptor sensitivity measure. One of the most used cardio-neural metrics is heart rate variability (HRV), a measure derived from the ECG that looks for dynamic heart rate changes attributable primarily to fluctuations in ANS activity [31]. HRV metrics can capture a breakdown in homeostasis due to either cardiovascular disease or cerebral disease; in fact, clinical activity or EDSS (Expanded Disability Status Scale) score and underlying inflammation are correlated with the alteration of HRV [26,32]. The sympathetic and parasympathetic system seems to be affected differently by MS. Parasympathetic dysfunction correlates with disease progression and disability, probably as a consequence of secondary degeneration [19]. Instead, sympathetic dysfunction correlates with clinical disease activity. Remarkably, vessels are innervated just by sympathetic systems, and vasomotor dysfunction has been described in patients with active disease compared to patients with stable disease [33,34]. Vasomotor alteration can contribute to the observed high prevalence of hypertension in MS patients (discussed below). It is crucial to note that sympathetic alteration can have a pathogenic role in MS; in fact, the sympathetic nervous system directly influences the immune system [32]. For example, noradrenergic fibers penetrate lymphoid organs and influence lymphocytes through contact and diffusible neurotransmitters. In addition, it impacts the circulating level of noradrenalin, which in turn modulates the proliferation of lymphocytes and the release of cytokines. Lastly, the sympathetic nervous system produces other neuropeptides (SP, SOM, NPY, VIP) that influence immune cells. For a more extensive discussion, see Benarroch et al. [22,35,36]. In favor of the pathogenic role of sympathetic alteration, Flachenecker et al. found lower median catecholamine levels in “active MS patients” than in those with stable disease. Moreover, in clinically active patients, sympathetic vasomotor dysfunction seems to be an early finding, more pronounced at baseline in active patients compared to stable patients [19]. 

In conclusion, sympathetic dysfunction deserves particular attention due to its possible role as a prognostic factor. An assessment of the ANS is not yet integrated into the clinical practice because more consistent data must be collected. Still, it could be reasonable to request autonomic testing in newly diagnosed patients or patients with fatigue and ANS-related symptoms. Patients with clearly pathological results should be carefully monitored because they can be at risk for more active disease and cardiovascular comorbidities, such as hypertension. Careful monitoring is essential for prompt treatment. Tilting and sudomotor testing are two available tests that can be useful in this context and have the advantages of being non-invasive and inexpensive.

## 2. Heart Health in MS Patients

### 2.1. Cardiovascular Risk in MS Patients

Chronic comorbidities are common in people with MS, contributing to worsening their prognosis and QoL and increasing the disease burden. Some authors reported an excess of mortality due to cardiovascular causes in MS patients compared to the general population [11,37]. A British study on cardiovascular risk events and mortality reported an almost 3-fold increased risk of all-cause mortality and a 1.5-fold increased risk of cardiovascular disease mortality in patients with MS [38]. Based on a systematic review, hypertension, hyperlipidemia, and diabetes are common in the MS population. The prevalence of the cited comorbidities increases with the patient’s age. [7,11]. An Italian observational study confirmed that the most common comorbidity among MS patients is hypertension, followed by diabetes, and noted that male MS patients are more affected by hypertension than the general population. The most important observation is that considering just young adults (20–44 years of age) of both sexes, MS patients had a higher rate of hypertension, stroke, and diabetes [39]; one possible explanation is the “surveillance hypothesis”. Anyway, hypertension and its origin in a young cohort deserve careful management and significantly dropped into the context of the general cardiovascular risk. 

To counterbalance a high cardiovascular risk, patients with MS must be empowered to adopt positive health behaviors. Smoking habits, obesity, and physical inactivity should be strongly discouraged in MS patients, although a healthy lifestyle is already suggested to MS patients given that smoking and being overweight or obese is recognized as an independent factor of a more rapid disability progression in MS [40,41] (see the section Discussion and clinical recommendations for more suggestion on the management of cardiovascular comorbidities). 

### 2.2. Cardiac Diseases in MS Patients 

Moving from risk factors to pathologies, two systematic reviews found an increased risk for myocardial infarction and heart failure in MS patients compared with the general population. Of note, a Danish study found a nearly two-fold increased incidence of heart failure (HF) in the incident MS population versus an age- and sex-matched cohort from the general population during the first year after MS diagnosis [42]. This finding suggests that heart damage may occur during the early phase of the disease, a stage characterized by relapses. In this early period, an altered circulation of immune cells and infiltration into the CNS parenchyma can be more present. 

Apart from disturbed cardiac autonomic control previously discussed, investigation of the heart in MS patients reveals an impaired left ventricular function; this happens more often than in healthy counterparts. It is important to define the nature of the cardiac alteration in order to plan an effective rehabilitation. According to a Cochrane review, there are moderate-quality pieces of evidence that both inpatient and outpatient can improve their disability thanks to multidisciplinary rehabilitation programs. Exercise and physical activities can improve mobility, muscular strength, QoL, patient-reported fatigue, and psychological symptoms, such as mood [43]. However, despite exercise’s cited benefits, cardiac autonomic control abnormalities are not easily remediated by exercise training [44]. For example, in healthy subjects, the heart rate increases significantly within the first 20 seconds of endurance exercises [45,46]. Still, the same does not happen in patients with MS even after training [47]. This alteration seems to have a central cause and correlate with walking capacity in patients [48]. This observation leads to the hypothesis that brain lesions or modifications of the cardiac tissue are among the causes of permanent alterations that persist despite therapeutical interventions [39,49]. It remains to be examined whether better cardiac functioning only occurs after selecting specific training modalities; for this reason, rehabilitation and training programs for MS patients may be currently suboptimal. On the other side, new cardiac symptoms in MS patients could be a sign of disease exacerbation. Acute cerebral disorders are well known to cause cardiac arrhythmias: hematoma, trauma, and hemorrhage, as well as direct stimulation of some areas, cause electrocardiographic abnormalities [50,51]. Acute CNS lesions can induce an increased release of catecholamines, causing necrotic changes in cardiac myocytes [52]. This, in return, can disrupt the endocardial conduction system causing arrhythmias [53]. Acute cardiac events have been observed in MS patients in relation to the progression of the disease: paroxysmal atrial fibrillation [54], cardiogenic shock [55], neurogenic pulmonary edema [56], and Takotsubo syndrome [57]. This latter represents approximately 1–6% of patients presenting with suspected acute coronary syndrome. Although its pathophysiology is not fully understood, excessive sympathetic system stimulation seems to play an important role [58,59,60]. Especially in recent years, reports of Takotsubo syndrome in MS patients are accumulating [61,62,63,64]. Of note, localization of lesions in critical areas of the CNS seems to be correlated with this acute cardiac presentation; in fact, in the described MS cases presenting as Takotsubo syndrome, lesions were located in the medulla oblongata involving the cardiovascular center, suggesting that damaging this zone leads to acute dysregulation of the cardiac sympathetic tone. The link between these two conditions is so convincing that some authors listed Takotsubo syndrome as an uncommon extra-neurological manifestation of MS [62]. Ultimately, excess mortality due to the cardiovascular cause can be ascribed to prolonged QTc interval reported in MS patients [29,65]. Such a measure is a predictor for unfavorable cardiovascular events, especially among hypertensive patients [66], but even in subjects without cardiac diseases [67].

Given the observed risk of cardiac diseases in MS patients, it could be useful to order ECG and echocardiography routinely. ECG should be performed annually. Moreover, even if it is not currently recommended, it is prudential to order echocardiography in the first year after the new diagnosis and after each relapse. In this way, we hope to obtain prompt recognition and treatment of cardiac conditions. 

## 3. Inflammation in MS and Heart 

### 3.1. Pathophysiology of Inflammation

Inflammatory and cardiac diseases, MS and myocarditis, respectively, show similarities in terms of molecular and immune mechanisms of the underlying histology and pathophysiology. Neurons, glial cells, and cardiomyocytes are all post-mitotic cells, and both the brain and the heart benefit from a relative immune privilege. MS and myocarditis are both focal diseases with characteristic histological lesions. The initial phases of MS are marked by active demyelinating plaques in the white matter following the breakdown of the BBB, possibly because of pro-inflammatory cytokines and chemokines. The demyelinating plaques show infiltration by T and B cells and activation of microglia, macrophages, and astrocytes, leading to loss of oligodendrocytes, reactive gliosis, and neuro-axonal degeneration [68]. MS patients with longer disease duration and with secondary progressive (SP) MS show chronic active lesions characterized by active macrophages at the edge of the lesion rather than at the center. On the contrary, progressive MS is usually characterized by inactive lesions with fewer lymphocytes [69]. 

In parallel, in myocarditis, we can find two stages of inflammation with different characteristics. Myocarditis is characterized by the presence of infiltrating inflammatory mononucleated cells, specifically >7 CD3+ T cells, and myocyte necrosis, with or without fibrosis [70]. After the acute phase, myocarditis can heal either with complete *restitutio ad integrum* or fibrotic scar. Alternatively, myocarditis can relapse or become chronic; in this phase, ongoing inflammatory processes and focal or diffuse fibrosis occur. Moreover, in chronic myocarditis, we assist in the development of myocyte structural abnormalities in the context of inflammatory cardiomyopathy and dilated cardiomyopathy (DCM) [71].

In MS, the most implicated effector T cells are CD4+ Th17, CD8+ T cells, as well as Th1 cells, with both peripheral and CNS-compartmentalized inflammatory mechanisms [72]. A specific subset of T CD4+ is also involved in myocarditis evolution. In particular, as demonstrated in men, cardiomyocyte damage, Th2, Th17, and Th1, resulted in promoting cardiac inflammation [73,74]. It seems that in human inflammation, the resolution is favored by the Th2 response [75,76]. The contribution of B cells in MS has been established, which are the target of anti-​CD20 biological drugs [77]. B cells might also contribute to sustaining chronic cardiac inflammation, leading to the development of DCM. However, circulating antibodies in patients with MS, including antibodies directed against myelin basic protein or myelin-oligodendrocytes glycoprotein, have not shown a clear pathogenetic role, in contrast with findings in neuromyelitis optica spectrum disorder (NMOSD) [78]. On the contrary, heart-specific autoantibodies were found in up to 60% of patients with myocarditis and were shown to target several cardiac autoantigens, such as cardiac α- and β-myosin heavy chains, with a possible direct pathogenic and/or prognostic role [79,80].

Another common feature of MS and myocarditis is that both are associated with viral infections. Although the precise mechanisms of tissue damage in multiple sclerosis and myocarditis are unclear, both viral replication and immune effector cells have been proposed to cause pathogenesis [75]. Notably, a solid association was recently confirmed between Epstein–Barr virus (EBV) infection and MS development [81]. A defective T cell control of EBV in patients with MS has been reported [82]. One proposed mechanism to explain EBV involvement in MS is molecular mimicry; another is the effect of EBNA-2 on B-cells. In fact, EBNA-2 inside the host cells binds within genetic loci associated with MS [83]. Nevertheless, myocarditis is most frequently caused by a viral infection, and human herpesviruses are also addressed as myocarditis-causative viruses [84]. In a case of chronic active myocarditis, EBV was found to induce a persistent infection of myocardial CD8+ T cells [85], while myocardial EBV infection caused acute myocarditis with heart failure, necrotizing coronary vasculitis, and multiple left ventricular aneurysms in another case [86]. In viral infections, Treg and Th17 cells can have diverse effects on viral infections ranging from exacerbating to preventing disease, and both have been proposed to be pathogenetic in MS and myocarditis [87]. For example, a reduction in Treg cells was observed in both MS and myocarditis [88,89]. Treatment that aims to restore the balance between Treg and Th17 cells may ameliorate viral pathology during infections. It is not surprising that glatiramer acetate, a drug yet used to treat MS, has been shown to modulate populations of Treg and Th17 cells, among other reagents [87]. However, we did not find reported cases of concomitant myocarditis, and MS. A possible explanation is that a single virus might be able to cause or exacerbate one of the two organ-specific immune-mediated diseases according to the subject’s genetic susceptibility [77,80,84,85,86]. Recently, acute myocarditis has been proposed as a rare complication in patients hospitalized for COVID-19, with an outcome that differs based on the presence of concomitant pneumonia [90,91]. The cardiac injury could be associated with critical illness and inflammation secondary to cytokine storm in patients with concomitant severe pneumonia. Still, coronaviruses alone could trigger harmful immune-mediated reactions in susceptible hosts with a permissive genetic background, even in the absence of critical respiratory disease [91]. Less severe myocarditis has also been associated with the anti-SARS-CoV-2 vaccine [92]. Regarding the relationship between MS and SARS-CoV-2, just a few cases of the new-onset disease in acute SARS-CoV-2 virus infection have been described [93,94,95]. However, coronaviruses have demonstrated neuroinvasive potential [96], and data show that SARS-CoV-2 infection can trigger both pseudo-relapses and true relapses [97]. Lately, vaccination seems to play a role in unmasking latent prior diseases, precipitating the first clinical event in the context of an exaggerated immune response [98]. 

### 3.2. MS and Myocarditis 

Despite the consistent parallels, the prevalence of overlapping MS and myocarditis is still to be investigated. As previously discussed, intrinsic cardiac dysfunction is reported in patients with MS, although sometimes not clinically manifest. MS patients have impaired biventricular function by comparison with normal subjects, with reduced left atrial function but normal arterial and endothelial function, suggesting an intrinsic myocardial disease [99]. Another study confirmed subclinical left ventricular dysfunction but found preserved right ventricular function in patients with MS [100]. However, whether inflammation was responsible for cardiac dysfunction was not assessed. We found a few case reports of myocarditis in patients with MS, but in each case, treatment could have a role in the development of the pathology. A case reported an acute necrotizing eosinophilic myocarditis diagnosed in a 31-year-old woman with a 4-year history of relapsing-remitting (RR) MS, under treatment with immunomodulating therapies, presenting with chest pain, biventricular function deterioration, and refractory ventricular tachycardia. Short courses of methylprednisolone infusions were given for relapses. Other medications in the past had included copaxone, amantadine, and daclizumab. She also was assuming chronically sertraline and oxcarbazepine and was allergic to amoxicillin and doxycycline. Three weeks before admission, her neurologist prescribed modafinil to treat the fatigue. After one week, she discontinued modafinil in favor of diphenhydramine because of the new appearance of pruritic rush and periorbital erythema [101]. The final diagnosis was “drug-induced hypersensitivity reaction with systemic extra-cardiac involvement”, and a definite relation to MS was excluded. However, several questions were raised about this case: the drug responsible for hypersensitivity was not certainly determined; virological analysis on plasma and cardiac biopsy was incomplete; positivity for antinuclear antibodies was significant; and chronic inflammation, specifically T CD4+, was found in the brain, spinal cord, meninges, and heart [102,103,104]. Another case of fatal giant cell myocarditis occurred in a patient with MS two years after alemtuzumab intake. It was deemed as a late immune-related adverse event of the therapy [105]. Of note, a case of enterovirus-related myocarditis in a patient taking rituximab to treat NMOSD was reported. The myocarditis was attributed to the immunocompromised status of the patient; in fact, he was B cell-depleted [106]. Remarkably, NMOSD was comorbid with myocarditis and myositis in a patient with antinuclear antibodies and anti-aquaporin 4 (AQP4) antibodies positivity [107]. For all these reasons, a direct link between demyelinating diseases and myocarditis cannot be excluded. 

Heart inflammation in MS patients is not a recognized characteristic of MS pathology, but it is probably underrecognized. More careful investigation of subtle cardiac alteration is recommended. Suppose the result of the ECG or the echocardiography is abnormal. In that case, further testing can be necessary according to the attending physician’s judgment; performing a blood test and measuring ESR, serum level of troponin, CRP, and NTproBNP, should be considered. An in-depth investigation can be completed with a cardiac MRI or biopsy if necessary. Since there are no strong recommendations on the topic, studies need to investigate the utility of such close cardiac monitoring. 

## 4. MS Therapy and Heart Consequences of Chronic Therapy

In recent years, MS treatment has benefited from the availability of new therapies, and many biological drugs can be prescribed now for these patients. Disease-modifying treatments (DMT) are those drugs that aim at reducing inflammation, disease activity, and all the long-term clinical consequences of the disease. DMT should be administered as soon as the patient has been diagnosed with MS, possibly making a distinction between the treatment for RRMS and progressive MS (PMS). Concerning RRMS, available drugs are divided into lines of treatment based on a trade of efficacy and adverse effect. The dominant current treatment strategy for RRMS is escalation therapy. First-line treatment drugs, including glatiramer acetate, INFβ, teriflunomide, and dimethyl fumarate, are safer but less effective. Fingolimod, alemtuzumab, cladribine, ocrelizumab, and natalizumab can be listed among the second-line therapy for RRMS. Rituximab and ocrelizumab should be considered to treat PMS, but also mitoxantrone was approved, and relapses are treated with high-dose corticosteroids [1]. Lately, new drugs have been approved for MS: FDA in 2020 approved ofatumumab for the treatment of adults with relapsing forms of MS, including CIS, RRMS, and active secondary PMS. Moreover, other drugs than fingolimod that target the sphingosine 1-phosphate (S1P) pathway have been approved, such as siponimod, ozanimod, and ponesimod. These drugs have different effects on S1P receptors. S1P receptors subtype specificity influences the downstream effects of the drug, including the aspects of the benefit-risk profile [108].

MS therapy itself can contribute to or cause cardiac pathology. Among the listed ones, mitoxantrone was the one with the clearest warning about cardiotoxic reactions [109,110]. For this reason, mitoxantrone is not commonly used in clinical practice, and other DMTs are preferred. However, MS patients treated with mitoxantrone should be periodically evaluated for left ventricular dysfunction [111]. Particular attention should also be paid to the use of high-dose corticosteroids, which are known to cause cardiac arrhythmias [112]. The start of the treatment with fingolimod is associated with a reduction in heart rate at the time of administration, and delayed fingolimod-associated asystole has been observed. Less commonly, a delay in atrioventricular conduction has been reported in some patients when initiating fingolimod [113,114,115]. Such adverse reaction of Fingolimod is due to activation of S1PR subtype 1 on cardiac myocytes. Fingolimod acts as a full S1PR agonist, which means it works as an S1PR antagonist after the downregulation of S1PR subtype 1 at the cell surface. There are 5 S1PR subtypes (S1P 1–5). S1P1, S1P2, and S1P3 are predominantly expressed in the cardiovascular, central nervous, and immune systems, whereas the expression of S1P4 and S1P5 is limited to the immune and central nervous systems, respectively [116]. As said before, new drugs that act in the S1P pathway have different receptor specificity; they are more selective than fingolimod and are associated with fewer adverse events. Regarding the first-line treatments for RRMS, glatiramer acetate is reported to induce hypertension, myocardial infarction, and increased risk of coronary artery disease [116]. Additionally, Teriflunomide can be associated with hypertension in treated patients; however, a positive benefit-risk profile is demonstrated in a long-term study [117]. On the opposite, dimethyl fumarate is protective against oxidative damage in heart cardiomyopathy and could improve left ventricular functioning, promoting reparative phenotype on cardiac myofibroblasts and macrophages [118,119]. Regarding the second-line treatments for RRMS, alemtuzumab is associated with several autoimmune phenomena and should be used only in cases where all other DMT are contraindicated or ineffective [119]. Finally, the two anti-CD20 drugs (ocrelizumab and ofatumumab) were also significantly associated with multiple cardiovascular adverse events; among all the cardiotoxic events, coronary artery disease, cardiac failure, and atrial fibrillation were the most predominant [120]. These data suggest further studies investigating the possibility of a more personalized prescription of these drugs in MS patients. Ideally, in the future cardiovascular risk profile should be taken into consideration. Before starting specific treatments, comorbid conditions should be considered in MS, as they can limit or negatively affect the utilization of some DMTs [39]. Nowadays, a cardiac evaluation is suggested before starting siponimod, but in the future, more features could be considered, such as repolarization time. Many patients have prolonged cardiac repolarization that increases during the disease course. Such a condition indicates bad cardiovascular outcomes and should be integrated with other information in choosing the optimal drug [65]. A summary of drugs and their cardiac outcomes is listed in Table 1.

## 5. Discussion and Clinical Recommendations

The spectrum of cardiovascular involvement in MS is wide and underappreciated. MS affects both the somatic and autonomic nervous systems. Autonomic dysfunction produces a serious impact on patient management. Assessing cardiovascular functioning is vital to reduce mortality, improve the QoL and plan optimal therapeutic strategies. Defining how MS lesions impact mortality other than neurological complications is important.

Given the high prevalence of hypertension, blood pressure should be checked at every clinic visit. In addition, patients with MS who are overweight or obese should be screened at least every three years for abnormal glucose levels. Performing a lipid screening at least every five years in adult patients with MS is also relevant. It is unknown whether treatment targets for diabetes, hypertension, and hyperlipidemia should differ in people with MS from those without MS; therefore, clinical trials are needed to test whether aggressive treatment of these conditions improves MS outcomes. Presently, at least primary prevention targets should be obtained. 

Another interesting point is that the inflammatory milieu observed in MS is very similar to that observed in myocarditis. Therefore, it will be very valuable to study such similarities in depth. These observations should prompt further research into cardiac inflammation in patients with MS, for example, through the screening of cardiac damage biomarkers (troponin, NTproBNP), imaging (echocardiogram, cardiac magnetic resonance), and histology (endomyocardial biopsy).

Moreover, a cardiac evaluation should be proposed even before starting a DMT. Most studies focused on mitoxantrone’s cardiotoxicity, which is now a reserved treatment. However, many of the available drugs still can have cardiac side effects. More information about patients’ cardiovascular risk should be obtained and integrated before starting a new drug to reach an optimal personalization of the therapy.

To date, the issue of cardiac health in MS patients deserves more emphasis. Currently, general considerations can be made to prevent cardiovascular comorbidities in the MS population, and their fulfillment should be implemented in clinical practice. Such aspects and other ones mentioned in this manuscript are summarized in Table 2.

## 6. Conclusions

In conclusion, there is little knowledge about cardiovascular involvement in MS. Research is required to improve our understanding and develop management algorithms capable of detecting, preventing, and treating cardiovascular adverse events in this population. A limitation of this review is that it is a narrative review, therefore no strict methodology has been used to select relevant studies and the quality of the cited studies has not been systematically assessed. Despite the limitations, the aim is to prompt further clinical and pre-clinical research on this topic. This is an important issue because MS is the leading cause of non-traumatic neurological morbidity; therefore, optimizing the medical management of these young, socially, and economically active patients is a priority.

## Figures and Tables

**Table 1:** Approved drugs for MS and cardiovascular effects.

Treatment	Type	Cardiac Effects (Incidence %)	Preventive Measures	Ref.
High dose corticosteroids	Anti-inflammatory and immunosuppressive	Arrhythmia, in particular sinus tachycardia (41.9%). In particular, women, smokers, elevated BMI, and other autonomic dysfunction	From observation and administration of pharmacological treatments based on ECG results up to temporary cardiac pacing. High-risk groups might benefit from cardiac Holter monitoring 12 h after the steroid pulse	[112]
Glatiramer acetate	s.c. synthetic polypeptides: shift of immune response	Hypertension, increased risk of coronary artery disease and myocardial infarction (rare), post-injection transient chest pain	Monitoring	[116]
Mitoxantrone	Anthracycline drug: ROS production, DNA intercalation	Early and late-left ventricular dysfunction (2%–4%)	Use of minimal effective dose and appropriate screening of cardiac function	[111]
IFN family	Recombinant protein	No evidence of cardiac effect	None	
Teriflunomide	Oral pyrimidine synthesis inhibitor	Hypertension (5.6%)	Monitoring	[117]
Dimethyl fumarate	Oral NRF2 agonist	Improves left ventricular functioning, promotes reparative phenotype on cardiac myofibroblasts and macrophages	None	[118]
Fingolimod	Oral S1P inhibitor	Symptomatic bradycardia (1%), delay in atrioventricular conduction (0.2–3%)	Monitoring for 6 h after the first dose. Concomitant use of heart rate-lowering drugs is not recommended	[113]
Alemtuzumab	i.v. monoclonal anti-CD52 antibody	Several autoimmune phenomena	Use when other DMT are contraindicated or ineffective	[120]
Cladribine	Oral purine analogue	No evidence of cardiac effect	None	
Ocrelizumab	i.v. monoclonal anti-CD20 antibody	Cardiotoxic events, coronary artery disease (0.76%), cardiac failure (0.28%), and atrial fibrillation (0.23%)	Obtain baseline electrocardiography and echocardiogram (need for cardiovascular risk stratification)	[120]
Ofatumumab	i.v. monoclonal anti-CD20 antibody	Cardiotoxic events, coronary artery disease (1.2%), cardiac failure (0.55%), and atrial fibrillation (1.07%)	Obtain baseline electrocardiography and echocardiogram (need for cardiovascular risk stratification)	[120]
Natalizumab	i.v. monoclonal anti-VLA4 antibody	No evidence of cardiac effect	None	

**Table 2:** Key clinical recommendations.

- Newly diagnosed patients should be tested for autonomic dysfunction, by tilting and/or sudomotor test
- Smoking habits, obesity, and physical inactivity should be strongly discouraged
- Blood pressure should be checked at every visit
- Glucose blood levels should be tested at least every 3 years
- Lipid profile screening should be performed at least every 5 years
- ECG should be performed annually, and echocardiography should be obtained in the first year from the diagnosis and eventually after relapses
- Cardiac inflammation should be investigated if suspected, in a research context, by dosage of cardiac enzymes (troponin, NTproBNP) and cardiac magnetic resonance
- Before starting a new drug and during follow-up, potential expected cardiovascular side effects should be screened and monitored
- Clinical trials to assess the usefulness of aggressive monitoring and treatment of cardiovascular comorbidities should be advocated

## Data Availability

No dataset were created and all considerations are available through bibliography.

## Abbreviations

MS	Multiple Sclerosis
CNS	Central Nervous System
BBB	Blood Brain Barrier
QoL	Quality Of Life
ANS	Autonomic Nervous System
CIS	Clinically Isolated Syndrome
HRV	Heart Rate Variability
EDSS	Expanded Disability Status Scale
HF	Heart Failure
SPMS	Secondary Progressive Multiple Sclerosis
DCM	Dilated Cardiomyopathy
NMOSD	Neuromyelitis Optica Spectrum Disorder
EBV	Epstein–Barr Virus
RRMS	Relapsing-Remitting Multiple Sclerosis
DMT	Disease-modifying Treatments
PMS	Progressive Multiple Sclerosis
S1P	Sphingosine 1-Phosphate
S1PR	Sphingosine 1-Phosphate Receptor
